# Impact of Circular Brewer’s Spent Grain Flour after In Vitro Gastrointestinal Digestion on Human Gut Microbiota

**DOI:** 10.3390/foods11152279

**Published:** 2022-07-30

**Authors:** Teresa Bonifácio-Lopes, Marcelo D. Catarino, Ana A. Vilas-Boas, Tânia B. Ribeiro, Débora A. Campos, José A. Teixeira, Manuela Pintado

**Affiliations:** 1 CBQF—Centro de Biotecnologia e Química Fina—Laboratório Associado, Escola Superior de Biotecnologia, Universidade Católica Portuguesa, Rua Diogo Botelho 1327, 4169-005 Porto, Portugal; tebonifacio@hotmail.com (T.B.-L.); avboas@ucp.pt (A.A.V.-B.); tribeiro@ucp.pt (T.B.R.); deborancampos@gmail.com (D.A.C.); 2 CEB—Centre of Biological Engineering, University of Minho, 4710-057 Braga, Portugal; jateixeira@deb.uminho.pt; 3 LAQV-REQUIMTE & Department of Chemistry, University of Aveiro, 3810-193 Aveiro, Portugal; mcatarino@ua.pt

**Keywords:** in vitro digestion, phenolic compounds, antioxidant activity, prebiotic activity, human gut microbiota

## Abstract

Brewer’s spent grain (BSG) solid residues are constituted by dietary fibre, protein, sugars, and polyphenols, which can have potential effects on human health. In this study, for the first time, the flours obtained from solid residues of solid-liquid extraction (SLE) and ohmic heating extraction (OHE) were applied throughout the gastrointestinal digestion simulation (GID), in order to evaluate their prebiotic potential and in vitro human gut microbiota fermentation. The results showed that the digestion of BSG flours obtained by the different methods lead to an increase throughout the GID of total phenolic compounds (SLE: from 2.27 to 7.20 mg gallic acid/g BSG—60% ethanol:water (*v/v*); OHE: 2.23 to 8.36 mg gallic acid/g BSG—80% ethanol:water (*v/v*)) and consequently an increase in antioxidant activity (ABTS—SLE: from 6.26 to 13.07 mg ascorbic acid/g BSG—80% ethanol:water (*v/v*); OHE: 4.60 to 10.60 mg ascorbic acid/g BSG—80% ethanol:water (*v/v*)—ORAC—SLE: 3.31 to 14.94 mg Trolox/g BSG—80% ethanol:water (*v/v*); OHE: from 2.13 to 17.37 mg Trolox/g BSG—60% ethanol:water (*v/v*)). The main phenolic compounds identified included representative molecules such as vanillic and ferulic acids, vanillin and catechin, among others being identified and quantified in all GID phases. These samples also induced the growth of probiotic bacteria and promoted the positive modulation of beneficial strains (such as *Bifidobacterium* spp. and *Lactobacillus* spp.) present in human faeces. Moreover, the fermentation by human faeces microbiota also allowed the production of short chain fatty acids (acetic, propionic, and butyric). Furthermore, previous identified polyphenols were also identified during fecal fermentation. This study demonstrates that BSG flours obtained from the solid residues of SLE and OHE extractions promoted a positive modulation of gut microbiota and related metabolism and antioxidant environment associated to the released phenolic compounds.

## 1. Introduction

Brewer’s spent grain (BSG) is a by-product from the brewing industry and due to its origin (malt grain husks with parts of the pericarp and seed coats of the barley), is an important source of bioactive compounds [1]. With the need to re-use by-products to create a new market value, BSG is an interesting by-product to take into consideration. BSG can be applied already in several different uses such as: paper manufacture, as a substrate, in nutrition, as an adsorbent, in the production of energy and charcoal and as a brick component [2]. The extracts from BSG have proven to be constituted, apart from polyphenols, a high content in dietary fibre and proteins, which promotes directly or indirectly the antioxidant capacity, anti-mutagenic, anti-microbial and anti-hypertensive activities [3,4,5].

There are several types of extractions able to obtain bioactive compounds from BSG, being solid-to-liquid extraction (SLE) and ohmic heating extraction (OHE) two efficient examples [2]. Solid-to-liquid extraction is both an eco-friendly and inexpensive method but optimal conditions need to be achieved [6,7,8] When applying OHE the electrodes contact the samples directly and the frequency applied is lower. The electric currents pass through the samples, and this makes the sample behave as a resistor in an electric circuit [9]. Since OHE is a clean technology, it needs a reduced energy cost, is less aggressive preventing the denaturisation of compounds, and it makes the cell more permeable which leads to the raise of phenolic compounds, but fouling deposits can occur and the processing degree can be limited by the density of the particles present in the extracts [9,10]. Both extractions typologies were able to extract soluble bioactive compounds from BSG (such as phenolic compounds) and the solid residues of the extractions proved to be still rich in dietary fibre and protein [3,11]. Nevertheless, SLE and OHE are recognised to be environment friendly technologies using as solvent ethanol and its mixtures and having low maintenance and energy consumption, preserving the molecular structure and activity [10,12,13,14].

The solid residues obtained after solid-to-liquid extraction after drying may constitute a high fibre ingredient with related health benefits. The extracts of these solid residues are already applied as food additives, in pharmaceutical industry, etc. However, to demonstrate the potential of the solid residues is critical to understand how digestion can affect the intrinsic molecules and how they become available to exert different beneficial effects. Simulation of the gastrointestinal intestinal digestion (GID) is one of the ways to study the bioaccessibility and digestibility of the bioactive compounds present in foodstuff and consequential impact on human health [15,16]. The GID simulation is composed of three phases: oral, stomachal and duodenal phases and the enzymes present in each phase are responsible for the breakdown of complex structure into small molecules [15,16,17]. However, some molecules are still not absorbed until the small intestine reaching the colon being exposed to the gut microbiota at the large intestine. Some of these components might exert a positive effect in the gut microbiota by modulating their growth as well as inducing the production of compounds such as short-chain fatty acids (SCFA). The SCFAs produced (acetate, butyrate and propionate) will be used from the colonic epithelium to the muscles, where they will play different functions with positive effects in health [18,19]. According to ISAPP consensus panel, “prebiotic” is “a substrate that is selectively utilised by host microorganisms conferring a health benefit’ [18,19,20,21]. Among other substrates inulin, FOS (fructooligosaccharides) and GOS (galactooligosaccharides), dietary fibres, have been already proved to be prebiotics. These dietary fibres comprise soluble (non-starch polysaccharides) and insoluble fibre (cellulose and hemicellulose) and can have attached antioxidants and proteins, which enable positive health effects. In addition, soluble dietary fibre can act as a promoter of prebiotic activity helping probiotic strains growth. Some studies report the digestion of dietary fibre linked with polyphenols forms bioactive metabolites that will increase the bioavailability, bioaccessibility and intake rates [15,18]. Some fractions of insoluble dietary fibre can be fermented by intestinal bacteria promoting the growth beneficial bacteria of *Bifidobacterium* and *Lactobacillus* genus, and the growth of *Bacteroides* and *Clostridium* (in a small extent), resulting in the formation of SCFA. However, there are studies showing that the effect that the dietary fibres can have on the bioactive compounds can be limited due to the pH temperature, polyphenol structure [22,23].

An integrated valorisation approach was made to BSG and the residue of the previous extractions procedures (obtained by SLE and OHE) proved to be rich mainly in dietary fibre. Taking all of this into consideration, the objective of this study was to perform GID simulation on BSG solid residues flour obtained through two extraction methods (SLE and OHE) and to compare the bioactivities associated to the flours—antioxidant and gut microbiota modulation. The total phenolics content and its quantitative profile, antioxidant activity achieved in each step of the GID (oral, gastric and small intestine phases) were evaluated to understand the impact on the BSG flours and compare the effect of the extraction method. Also, the prebiotic activity of the solid residues was also tested upon four types of probiotic bacteria: *Bifidobacterium animalis* B0, *Bifidobacterium animalis* spp. *lactis* BB12, *Lacticaseibacillus casei* 01 and *Lactobacillus acidophilus* LA-5. To validate the potential modulatory effects towards the gut microbiota, fermentation human faces model was used to demonstrate the capacity to modulate key groups of microorganisms and the related metabolic activity.

## 2. Materials and Methods

### 2.1. Raw Material

Brewer’s spent grain was dried for 24 h at 60 °C using a convective oven (Memmert, Schwabach, Germany) and up to 90% of dried matter. The BSG was vacuum stored at room temperature. Two lots were mixed to obtain a representative and homogeneous sample. Sample was supplied by Super Bock Group (Leça do Balio, Portugal).

### 2.2. Extraction Procedure

Before GID, BSG extractions were performed with absolute ethanol (C_2_H_5_OH, (Carlo Ebra, Sabadell, Spain) and water (H_2_O) mixtures (ethanol at 80% (*v/v*) and 60% (*v/v*)).

When using SLE, the extraction procedure was made according to [3] with some modifications. Briefly, dried BSG was mixed with solvent (20 g to 100 mL) in 150 mL flasks. An Ultraturrax T18 (Ika, Germany) was used to homogenise for 2 min at 1900 rpm and the flasks were stirred for 30 min to promote the extraction. Centrifugation at 2330× *g* for 10 min and room temperature (Hettich Zentrifugen Universal 32R, Germany) was made, and a 4–7 µm filter (Prat Dumas, Couze St. Front, France) was used to filter. Ethanol was extracted using a rotary evaporator (175 mbar at 40 °C) (Buchi, Switzerland). The solid residues were dried up to 90% of dry matter at 60 °C for 24 h and stored in vacuum. All extractions were done in triplicate.

When using OHE, the extraction procedure was made according to [24]. A jacketed static ohmic heater with a volume of 500 mL was used and the voltage controlled using a generator (Agilent 33.220 A, Bayan Lepas, Malaysia, 1 Hz–25 MHz, and 1–10 V) working with a sinusoidal wave at 25 kHz connected to an amplifier (Peavey CS3000, Meridian, MI, USA, 0.3–170 V). A thermocouple k-type (precision temperature ±1 °C, Omega, Stamford, CT, USA) was placed at the geometric centre of the sample volume and was then connected to a data logger (USB-9161, National Instruments Corporation, Austin, TX, USA). A constant 8 cm was kept as a distance between the electrodes. Heating rates were maintained through the extractions using a circular water system (F25-ED, Julabo, Seelbach, Germany). Electrical frequency, current intensity and voltage were measured through a portable oscilloscope (ScopeMeter^®^ 125/S, Fluke, Everett, WA, USA). After filtration, ethanol extraction and drying of the residues were made as for SLE. The temperature of the extractions was of 35 °C.

### 2.3. Simulated In Vitro Gastrointestinal Tract

The GID was made according to the one described [15]. BSG flours (1 g) were added to 20 mL of distilled water. Aliquots of 2 mL were taken for the initial values of antioxidant activity, total phenolic content and phenolic compounds. The pH in all samples was adjusted to pH values between 5.6 and 6.9 using NaHCO_3_ (1 mol/L) (Merck, Darmstadt, Germany).

#### 2.3.1. Oral Digestion

A solution of α–amylase (100 U/mL) (Sigma, Darmstadt, Germany) was added at a rate of 0.6 mL/min of digestion. The solutions were incubated in a shaking water bath for 2 min, at 37 °C at 200 rpm.

#### 2.3.2. Gastric Digestion

After, for gastric digestion, the pH was adjusted to 2.0 using HCl (1 mol/L) and pepsin (from porcine stomach mucosa, pepsin A 250 U/mg) (Sigma, Darmstadt, Germany) was added (25 mg/mL at a rate of 0.05 mL/mL of sample). Solutions were incubated in a shaking water bath for 120 min, at 37 °C at 130 rpm.

#### 2.3.3. Intestinal Digestion

In the end, intestinal digestion was performed by adjusting the pH to 6.0 using NaHCO_3_ (1 mol/L) (Merck, Darmstadt, Germany) and by the addition of 2 g/L of pancreatin (from porcine pancreas 8 × USP) (Sigma, Darmstadt, Germany) and 12 g/L of bile salts (Sigma, Darmstadt, Germany) at a ratio of 0.25 mL/mL of sample. The solutions were then incubated in a shaking water bath for 120 min at 37 °C, at 45 rpm.

Aliquots of 2 mL were taken between each step of the digestion for further analyses.

#### 2.3.4. Antioxidant Activity

##### ABTS Radical Cation Assay

The 2,2-azinobis-3-ethylbenzothiazoline-6-sulphonic acid (ABTS+) method was based on the method described by Gião et al. (2007) [25] modified to microplate [26]. The free radical-scavenging activity of the extracts was determined by this method. Ascorbic acid (C_6_H_8_O_6_) (Sigma, Darmstadt, Germany) at different concentrations (50.00–500.00 µM) was used as a standard curve and results were expressed as mg of ascorbic acid equivalents and all the assays were performed in triplicate.

##### Oxygen Radical Absorbance Capacity (ORAC)

The ORAC method was performed according to the one described by Contreras et al. (2011) [27]. The sample (at different concentrations), or an antioxidant [trolox (C_14_O_18_H_4_) (Sigma, Darmstadt, Germany) (0.0025 mg/mL–0.02 mg/mL)], fluorescein (C_20_H_12_O_5_) (Sigma, Darmstadt, Germany) (116.6 nM), 2,2′-Azobis (2-methylpropionamidine) dihydrochloride (AAPH) (Sigma, Darmstadt, Germany) (14 mM) were mixed with 75 nM of phosphate buffer (pH 7.4). The assay was performed at 40 °C and the fluorescence was read during 137 min (104 cycles). Multidetection plate reader (BioTek Synergy H1, Vermont, VT, USA) with two filters (485 nm excitation and 520 nm emission) was used to read the results of the microplate. Trolox and AAPH solutions were prepared daily, and fluorescein was diluted from a stock solution (1.17 mM) in 75 mM phosphate buffer (pH 7.4). The results were expressed as mg of trolox equivalent, and all analyses were performed in triplicate. The percentage of the differences between the different phases of the GID simulation was also calculated for the ORAC results.

##### Total Phenolic Content

Total phenolic content was determined by using the method Folin–Ciocalteu (Merck, Darmstadt, Germany) method previously described by Vilas-Boas et al. (2020) [26] adapted to microplate and was based on the one described by Gao et al. (2000) [28]. All analyses were performed in triplicate and a calibration curve of gallic acid (C_7_H_6_O_5_) (Sigma, Darmstadt, Germany) standard solutions (different concentrations from 0.04 to 0.20 mg/mL) was used to calculate total phenolic content which was calculated as gallic acid equivalent. The percentage of the differences between the different phases of the GID simulation was also calculated for the total phenolic content results.

##### Determination of Polyphenolic Profile and Composition

Phenolic content was characterised by HPLC during GID and after gut microbiota assays using the method previously described by Bonifácio-Lopes et al. (2020) [3]. A Kromasil 100-5-C18 column (250 mm–4.6 mm) (Bohus, Strömstad Sweeden) equipped with a C-18 precolumn was used and the PDA acquisition wavelength was set from 212 to 600 nm. Stationary Phase: Kromasil 100-5-C18 column (250 mm–4.6 mm). Mobile Phase: Solvent A: acetonitrile (100%) (Merck, Darmstadt, Germany) (CH3CN) with 0.1% TFA; Solvent B: acetonitrile/water (5:95 *v/v*) with 0.1% trifluoroacetic acid (TFA) (Sigma, Darmstadt, Germany) (CF3COOH); flow rate of 1 mL/min. The following gradient was employed: 0–2 min (100% B); 2–28 min (60% B); and 28–30 min (100% B). Standards solutions with concentrations range from 0.1000 to 0.0004 mg/mL were used for the identification and quantification of the following compounds: 3-5 dimethoxy-4-hydroxybenzaldehyde (98% purity) acid, 4-hydroxibenzoic acid, caffeic acid, catechin, ferulic acid, p-coumaric acid, protocatechuic acid, syringic acid, transcinnamic acid, vanillin, vanillic acid (Sigma, Darmstadt, Germany) and hydrocinnamic acid (Extrasynthese, Genay, France) expressed as micrograms per g of BSG. All calibration curves were linear over the concentration ranges tested, with correlations coefficients of 0.999. All assays were performed in triplicate.

##### Bioaccesibility Index

To determine the effect of GID on the polyphenols present in BSG flours, bioaccessibility index (B%) was used. The initial (before digestion) values were considered as 100% for all the compounds. Bioaccessibility index determines the amount of each polyphenol identified initially and in the end of the GID following Equation (1):

Bioaccessibility Index (%) = BiPC/Biinitial × 100(1)

where BiPC is the bioactive compound (mg/mL BSG) in the fraction of each GID simulation and Biinitial is the bioactive compound (mg/mL BSG) quantified in the initial BSG solid extract.

### 2.4. Prebiotic Activity

Bifidobacterium animalis B0 (CSK, Ede, Netherlands), Bifidobacterium animalis spp. lactis BB12, Lacticaseibacillus casei 01 and Lactobacillus acidophilus LA-5 (Chr. Hansen, Hørsholm, Denmark) were used to determine the prebiotic potential of the BSG flours. The strains were stored at −80 °C in MRS broth (Biokar Diagnostics, Beauvais, France) with 30% (*v/v*) of glycerol. Bacterial colonies of L. casei 01 and L. acidophilus LA-5 were suspended in MRS broth, achieving a turbidity of 0.5 McFarland and diluted to reach the recommended concentration of prebiotic bacteria of 5 × 105 CFU/mL. The inoculums were then added (20 µL) to a 96-well microplate and the BSG flours were added, diluted in basal MRS broth without glucose, to the microplate wells at concentrations of 1, 1.5 and 2% (*v/v*). After, the microplate was incubated (Multiskan GO, Thermo Scientific, Waltham, MA, USA) for 48 h, at 37 °C with agitation. Additionally, B animalis B0 and *B. lactis* BB12 inocula were prepared under anaerobic atmosphere suspending each colony in MRS broth supplemented with 0.05% (*v/v*) of L-cysteine-HCl, achieving a final turbidity of 0.5 McFarland standard, and, after diluted to reach the recommended concentration of prebiotic bacteria in wells of 5 × 105 CFU/mL. The inoculum was then added (20 µL) to a 96-well microplate and the BSG flours were added, diluted in basal MRS broth without glucose, to the microplate wells at concentrations of 1, 1.5 and 2% (*v/v*). The microplate was sealed with paraffin and incubated (Multiskan GO, Thermo Scientific, Waltham, MA, USA) during 48 h for B animalis B0 and *B. lactis* BB12 and 26 h for *L. casei* 01 and *L. acidophilus* LA-5, at 37 °C with agitation.

In both cases OD measurements were made every hour at 620 nm.

Negative control was only MRS broth and the positive controls were the inoculum at MRS broth supplement with glucose and MRS broth supplement with FOS.

### 2.5. In Vitro Fermentation Assays

Sterile plastic vases were used to collect human faeces and were kept in anaerobic conditions until further use (maximum of 2 h after being collected). Fresh samples were obtained from healthy human donors that did not have any known metabolic and/or gastrointestinal disorder, as well as not be taking any probiotic or prebiotic supplements or antibiotics for the last 3 months. The basal medium was prepared as described by Madureira et al. (2016) [29], being a nutrient base medium (5.0 g/L trypticase soya broth (TSB) without dextrose (BBL, Lockeysville, MD, USA), 5.0 g/L bactopeptone (Amersham, Buckinghamshire, UK), 0.5 g/L L-cysteine-HCl (Merck, Germany), 1.0% (*v/v*) of salt solution A (100.0 g/L NH_4_Cl, 10.0 g/L MgCl_2_·6H_2_O, 10.0 g/L CaCl_2_·2H_2_O), 0.2% (*v/v*) of salt solution B (200.0 g/L K_2_HPO_4_·3H_2_O) and 0.2% (*v/v*) of 0.5 g/L resazurin solution), prepared in distilled water and with pH of 6.8. This basal medium was poured into airtight glass anaerobic bottles, sealed with aluminium caps and then were sterilised by autoclave. Syringe filters of 0.2 μm (Chromafils, Macherey-Nagel, Düren, Germany) were used to sterilise stock solutions of Yeast Nitrogen Base (YNB) and inserted into the bottles. The serum bottles were incorporated with the BSG flours of SLE and OHE extractions hoven dried after GID simulation at a final concentration of 2% (*w/v*) and inoculated with faecal slurries of 2% (*v/v*) at 37 °C for 48 h without shaking or pH control. At 0, 12, 24 and 48 h of fermentation samples were taken. All the experiments were carried out inside an anaerobic cabinet with 5% of H_2_, 10% of CO_2_ and 85% of N_2_ and performed in compliance with the institutional guidelines.

### 2.6. Gut Microbiota Evaluation

#### 2.6.1. DNA Extraction

Extraction and purification of genomic DNA from stool samples was made as previous described by Campos et al. (2020) [15] using NZY Tissue gDNA Isolation Kit (Nzytech, Lisboa, Portugal) with some modifications. Samples were centrifuged to separate the supernatant from the pellet at 11,000× *g* for 10 min. After, around 170–200 mg of pellet was taken from control and test samples for all times. After, the pellets were washed with TE buffer (10 mM Tris/HCl; 1 mM EDTA, pH 8.0), vortexed and centrifuged again at 4000× *g* for 15 min, supernatant was discarded and 180 μL of a freshly prepared lysozyme solution (10 mg mL^−1^ lysozyme in NaCl-EDTA solution; 30 mmol L-1 NaCl and 10 mM EDTA) was added to the pellet. Then, pellet was incubated during 1 h at 37 ± 1 °C with periodic shaking to guarantee the total breakdown of the bacterial cell wall to improve DNA extraction efficiency. Afterwards, 350 μL of buffer NT1 were added to the samples. The samples were then vortexed and incubated at 95 °C for 10 min and centrifuged at 11,000× *g* for 1 min. Proteinase K (25 μL) was added to 200 μL of supernatant and incubated at 70 °C for 10 min. The remaining steps followed the manufacturer’s instructions. Purity and quantification of DNA were assessed with a NanoDrop spectrophotometer (ThermoScientific, Wilmington, DE, USA).

#### 2.6.2. Real-Time PCR for Microbial Analysis at Stool

For the real-time PCR the method was performed as described by Campos et al. (2020) [15] using sealed 96-well microplates, a LightCycler FastStart DNA Master SYBR Green kit and a LightCycler instrument (Roche Applied Science, Indianapolis, ID, USA). The PCR reaction mixtures (a total of 10 μL) consisted of 5 μL of 2 × Faststart SYBRGreen (Roche Diagnostics Ltd.), 0.2 μL of each primer (at a final concentration of 0.2 μM), 3.6 μL of water and 1 μL of DNA (equilibrated to 20 mg). The primer sequences (Sigut microbiota-Aldrich, St. Louis, MO, USA) used to target the 16S rRNA gene of the bacteria and the conditions for PCR amplification reactions are described in Table 1.

To verify the specificity of the amplicon, a melting curve analysis was performed via monitoring SYBR Green fluorescence with a temperature ramp of 60 to 97 °C. LightCycler software (Roche Applied Science) was used to process and analyse the data. Standard curves were constructed using serial tenfold dilutions of bacterial genomic DNA, according to the following webpage (http://cels.uri.edu/gsc/cbdna.html (accessed on 1 June 2020)). Bacterial genomic DNA used as a standard (Table 1) was obtained from DSMZ (Braunschweig, Germany). Genome size and copy number of the16S rRNA gene for each bacterial strain used as a standard was obtained from NCBI Genome database (http://www.ncbi.nlm.nih.gov (accessed on 1 June 2020)). Data are presented as the mean values of duplicate PCR analyses. The F:B ratio was obtained by dividing the number of copies of Firmicutes divisions by the number of copies of Bacteroidetes divisions. Moreover, the relative differences to negative control percentage (only faeces fermentation) were calculated using the following equation:

Relative difference to control %=(SMC−CMC)/(CMC)×100

where *SMC* is the mean copy numbers of the sample at a certain time (12 or 24 or 48 h) and *CMC* is the mean copy numbers of the control sample at the same time as *SMC*. Positive % values mean the occurrence of an increase in the number of copies relative to the control sample at that certain time. Higher the value, the higher increase.

#### 2.6.3. Determination of Organic Acids

Supernatants from the batch cultures were filtered through 0.20 μm cellulose acetate membranes. The chromatographic analysis was performed using a Beckman & Coulter 168 series HPLC system coupled with an UV and RI detector (Knauer, Berlin, Germany). The separation was performed using Aminex HPX-87H column (BioRad, Hercules, CA, USA) operated at 55 °C using A mobile phase, 0.003 mol/L H_2_SO_4_; flow, 0.6 mL/min. Aliquots of the filtered samples were assayed for organic acids (lactic, acetic, succinic, propionic and butyric) and the identification and quantification were achieved by comparison of the relative retention times of sample peaks with standards and using a calibration curve in the range of concentrations of 0.2–2.0 mg mL^−1^.

### 2.7. Statistical Analysis

The significance of the differences between different stages of GIT digestion was determined by one-way analysis of variance (ANOVA) according to the normality of data distribution (Shapiro-Wilk test) at the *p* < 0.05. The homogeneity of variances was assessed by Levene’s test, and the multiple comparisons were made at those statistically significant variables using the Tukey’s posthoc test at the *p* < 0.05 significance level. In cases where normality was not ensured (organic acids, sugars and polyphenols profiles during gut microbiota fermentation), non-parametric tests were used and null hypothesis that means are equal was rejected when the difference between means was *p* < 0.05. All the statistical analyses were performed using IBM SPSS Statistics (version 23, IBM, Armonk, NY, USA).

## 3. Results and Discussion

### 3.1. Bioaccessibility under Gastrointestinal Conditions

#### 3.1.1. Content and Profile of Phenolic Compounds

##### Total Phenolic Content

Throughout the GID simulation of BSG extracts’ flours, the total phenolic content (TPC) was measured using the Folin-Ciocalteau method and results are present in Table 2.

In light of the initial samples affected by the oral phase, it is possible to observe a decrease in TPC in BSG flours obtained when using both extraction methods (SLE and OHE) and solvents (60 and 80%). In this stage the range of difference of percentage between the phases was between 79% and 81%, and there were no significant differences between methods, with the exception between 60% ethanol:water (*v/v*) SLE and 80% ethanol:water (*v/v*) OHE, being the first one the one with the highest TPC in this phase of the GID. After this initial loss, the GID influenced positively the TPC, which meant that the TPC increased in the stomach and duodene phases of the GID simulation, increasing between 1 and 2 times.

As stated above, in the oral phase, 60% ethanol:water (*v/v*) SLE had the highest TPC value; however, there was no statistically difference between this extraction and the 60% ethanol:water (*v/v*) OHE. From the oral phase to the stomachal phase there’s an increase in the TPC values for all the extractions. Here, there’s significant differences between the extractions with the exception of 80% ethanol:water (*v/v*) SLE and 60% ethanol:water (*v/v*) OHE and these two were the extractions with higher values of TPC. From the stomach phase to the duodenal phase there was also an increase in all the tested extraction methods and in this phase the method with higher TPC was the 80% ethanol:water (*v/v*) OHE. Bound phenolics are normally higher in cereals than in the free form and can only be extracted when using acid or base hydrolysis [30].

From the mouth phase to the duodenal phase the TPC increased, having a higher increase in the last phase (small intestine phase). This might happen throughout the GID not only because the polyphenols were digested, but the proteins and fibres present were also digested. This digestion might help the release of the polyphenols, which might be linked to these compounds as some polyphenols are strongly bounded to other compounds thus taking more time to be released. Taking this in consideration there will be a release of small molecular weight polyphenols during the oral phase, while the ones with high molecular weight are going to take more time being released, this happening in the late stages of the GID [15,31,32,33,34].

In this study there was a drop of TPC from the initial to the oral phase and an increase in TPC throughout the GID. However, Chan et al. (2012) [30] showed that the TPC of wheat and rice flours was higher after GID simulations than in the initial samples. Various factors might influence the TPC results during GID such as the solvent used, the extraction method used and the polyphenols present in the raw material [35].

##### Individual Phenolic Compounds

Brewer’s spent grain is a good source of polyphenols due to its composition as BSG is mainly composed of grain husks combined with parts of the seed and pericarp of barley malt. Ferulic and *p*-coumaric are some of the polyphenols that can be present in BSG, but others as 4-hydroxybenzoic, vanillic, gallic and syringic acids and catechin and vanillin. The concentration of these compounds can vary depending on the beer production, as well as, a kind of malt used and production typology [1,3,6,24,26,36,37,38].

When polyphenols are released from the matrix during gastrointestinal digestion they become bioaccessible and, therefore, they are able to exert their bioactivity. For this reason, bioaccesibility is the percentage of a type of polyphenol released from the matrix and that becomes available for absorption in the gut [26]. In Table 2 it is possible to observe the various polyphenols present during the GID simulation of the BSG flours. Vanillic and ferulic acids (hydroxycinnamic acids), 4-hydroxybenzoic and *p*-coumaric acids (hydroxybenzoic acids), vanillin (benzaldehyde) and catechin (flavanol).

As BSG is rich in ferulic and *p*-coumaric acids these were expected to appear in higher concentrations. By observing Table 2 for the highest concentration is for ferulic acid in both extraction methods and for *p*-coumaric acid when using SLE 60% ethanol:water (*v/v*).

With regard to BSG flour obtained by SLE, the major polyphenols present were catechin, and 4-hydroxybenzoic acids, while in BSG flour obtained by OHE the major polyphenols present were catechin and ferulic acid. It was also possible to observe that the OHE lead to flour with higher concentration of polyphenols present during the GID and the highest bioaccesibility percentages. Wth regard to *p*-coumaric, this compound was present in higher concentrations in 80% ethanol:water (*v/v*) OHE and vanillin in 60% and 80% ethanol:water (*v/v*) OHE.

In Table 2 it was possible to observe that there was a decrease in all individual polyphenols in flours when exposed to the oral phase. This decrease continues throughout the GID with the exception of vanillin in the OHE where it was observed an increase in its concentration in the stomachal and intestinal phases. As the concentration of the polyphenols present decreased during the GID simulation, it might mean that the GID degraded the molecules making them loose their activity. Some polyphenols (as hydroxycinnamic acids) can be present in fibre linked to polysaccharides of the cell wall, being slowly released during the GID simulation; however, this did not occur. In this study, compounds normally related to insoluble dietary fibre (vanillic and 4-hydroxycinnamic acids) were not present during the GID simulation, which might mean that the GID was not capable of releasing them and thus were not absorbed by the small intestine. The ones identified during the GID (as *p*-coumaric 80% ethanol:water (*v/v*), catechin (in all the residues), and vanillin (60% and 80% ethanol:water (*v/v*), being now released and bioaccessible for absorption in the intestine might be associated with the soluble dietary fibre [15,26,39].

The loss of polyphenols during the GID simulation is well documented [40,41,42,43]. As these compounds are sensitive to the pH in the small intestine, some polyphenols will be degraded or transformed into other compounds [44] (Gayoso et al., 2016). However, there are studies that report polyphenol stability after GID simulation [45,46,47,48]. One thing that might help to explain the differences obtained between results are associated to the loss of chemical structure of the polyphenols recovered during the different stages of the GID simulation. These polyphenols recovered belong to four different groups: hydroxycinnamic and hydroxybenzoic acids, flavanols and benzaldehyde. Their solubility normally increases with water in proportion with the number of hydroxyl groups present in the molecules and, these compounds are, normally, also soluble in polar solvents [49]. The food matrix and the type of simulation used will also have an effect on the recovery of the polyphenols present [45].

### 3.2. Antioxidant Activity

#### ABTS and ORAC

**Figure 1:**
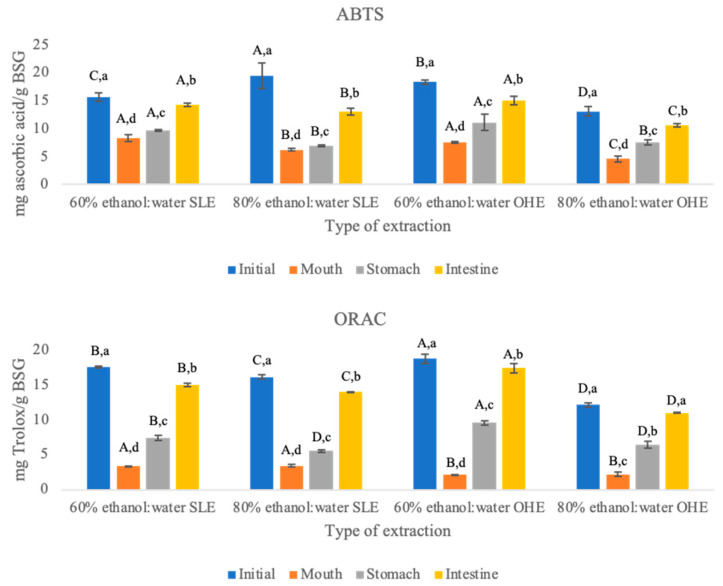
ABTS and ORAC values of brewer’s spent grain solid residues (from solid-liquid extraction (SLE) and ohmic heating extraction (OHE)) throughout GID. Different letters mean significant differences (*p* < 0.05). The capital letters indicate the differences among the different extracts at the same GID phase, and the lowercase letters indicate the differences between GID phases for each residue of extraction.

Antioxidant activity of BSG flours from SLE and OHE was performed throughout the GID and results are shown at Figure 1. From the initial values to the oral phase, it is possible to observe that there was a decrease in the antioxidant values in both methods (Table 1) (between 47 and 57% for ABTS/SLE, 58 and 64% for ABTS/OHE, and 79 and 81% for ORAC/SLE, 81 and 88% for ORAC/OHE—Table 3), but after that, the antioxidant activity in both methods increased in the other GID simulation phases.

The two antioxidant methods used in this study use different mechanisms to evaluate the antioxidant potential. ABTS assay uses an electron transfer method, where a potent antioxidant will transfer one electron that will reduce radicals—this capacity is measured. On the other hand, ORAC assay measures a hydrogen atom transfer reaction—the antioxidant is able to quench free radicals while using hydrogen donation, the radical source used by the ORAC method is H_2_O_2_, resembling one of the most representative biologically source [18,50]. For this reason, the new oxidation products that were formed in the GID when measured by the ORAC method might have an antioxidant potential lower than the ones present in the initial phase. Even though ORAC only measures the antioxidant activity when hydrogen transfer occurs, most likely the oxidation products formed during the GID simulation are able to quench radicals by single election transfer, as normally deprotonation will increase the electron-donating capacity of polyphenols [51,52].

Throughout GID and regarding ABTS method it was possible to observe that the extraction using 60% ethanol:water (*v/v*) was consistently the extraction that had higher antioxidant activity values in both SLE and OHE. Values between 60% ethanol:water (*v/v*) from SLE and 60% ethanol:water (*v/v*) from OHE are similar in oral, stomach and in duodenal phases (no statistical difference (*p* < 0.05). When comparing 80% ethanol:water (*v/v*) from SLE with 80% ethanol:water from OHE antioxidant activity values measured through ABTS were statistically different (*p* < 0.05) throughout the GID with the exception of stomach. The increase in ABTS antioxidant activity between oral phase and intestine phase was higher when OHE was applied and the solid residue of the extract using 80% ethanol:water (*v/v*) was the one with higher percentage of increase. The reason why 60% ethanol:water (*v/v*) was the extraction with higher antioxidant activity values might be because of the higher polarity of this mixture as the addition of water to organic solvents increases the polarity of the medium, which in turn might help facilitate the extraction of polyphenols, and in turn the highest antioxidant activity [53,54].

When using ORAC as an antioxidant activity measurement method it was also possible to observe that the increase between phases is higher than when using ABTS as an antioxidant activity measurement method. Looking at Figure 1 it is possible to observe that the antioxidant activity also increases throughout the GID. In the ORAC method there was also an initial decrease and in the oral phase there is no statistical difference (*p* < 0.05) between within the extraction method. After this decrease there was an increase in the other two phases of the GID. In these two phases the differences in the results are statistically different (*p* < 0.05). Studies of Gayoso et al. (2016) [45] and Tarko et al. (2009) [55] observed that the antioxidant activity is closely dependent of the concentrations of total phenolic compounds [55].

In this study, from the beginning of GID (oral phase) to the end (duodenal phase) the antioxidant activity of all the samples increased in both types of extraction (SLE and OHE). In other studies, the gastrointestinal tract has also proven to help increase the antioxidant activity of cereal products. The digestion of proteins and other compounds might also help to increase the release of polyphenols [1,31,32,33,34]. The increase in the antioxidant activity when using both methods throughout the GID can be linked to the fact that the polyphenols can be linked to soluble dietary fibre. Some polyphenols as hydroxycinnamic acids have higher molecular weight and have a stronger bound to dietary fibre that can make them take more time to being released during digestion. There has also been a report of a direct connection between the increase in the antioxidant activity and increase in the polyphenolic content. During the oral phase there is a release of smaller molecular weight polyphenols, while the ones with higher molecular weight are released in late phases of the GID [15].

In literature, there is not a consistency in the results published. While Carbonell-Capella et al. (2015) [56], Celep et al. (2015) [40], Rodríguez-Roque et al. (2013) [57], Ribeiro et al., 2020 [18], Rodríguez-Roque et al. (2014) [58] reported loss in antioxidant activity of polyphenols during GID, in accordance to the results obtained in this study, Campos et al. (2020) [15], Oliveira and Pintado (2015) [59], Pineda-Vadillo et al. (2016) [51] and Tagliazucchi et al. (2010) [48] reported an increase in the antioxidant activity of polyphenols during GID. The differences between the results can be related to the fact that some products formed during the GID might have higher antioxidant activity than their precursors, or to the fact that the alkaline intestinal environment can develop deprotonation of the hydroxyl moieties of the aromatic ring of the phenolics [45].

The impact of GID upon flours resulting from the BSG flours during GID was made for the first time in this study, so there was not a possibility of making a direct comparison. The increase of antioxidant activity throughout the GID also happened with Chan et al. (2012) [30] and Xia et al. (2020) [50], both when using wheat and barley products.

### 3.3. Prebiotic Activity

**Figure 2:**
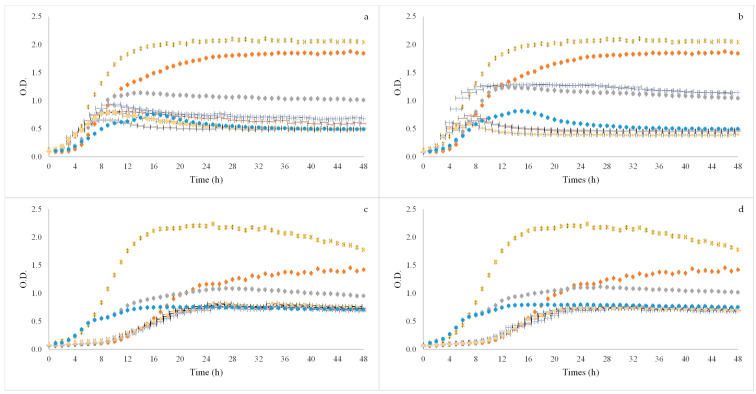
Growth curves of probiotic microorganisms *Bifidobacterium animalis B0* (**a**,**b**) and *Bifidobacterium animalis* spp. *lactis* BB12 (**c**,**d**) after applying BSG flours of SLE (**a**,**c**) and OHE (**b**,**d**) using three concentrations (2, 1.5 and 1% (*v/w*). x Positive control—Glucose; ● Positive control—FOS; + 60% ethanol:water SLE or OHE_2% (*v/w*); - 60% ethanol:water SLE or OHE_1.5% (*v/w*); ● 60% ethanol:water SLE or OHE_1% (*v/w*); - 80% ethanol:water SLE or OHE_2% (*v/w*); 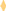 80% ethanol:water SLE or OHE_1.5% (*v/w*); ● 80% ethanol:water SLE or OHE_1% (*v/w*).

**Figure 3:**
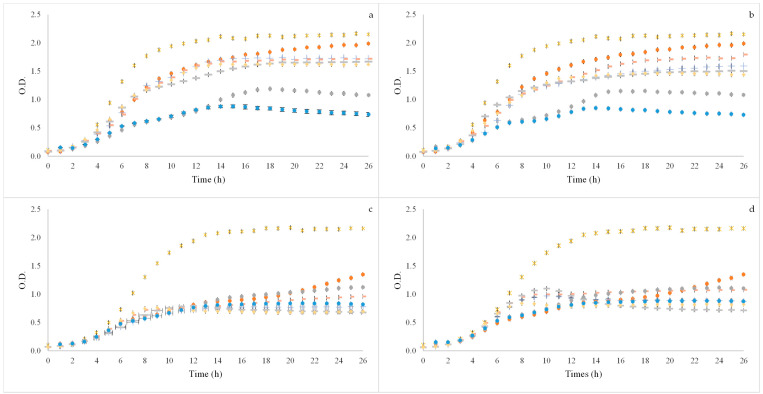
Growth curves of probiotic microorganisms *Lacticaseibacillus casei* 01 (**a**,**b**) and *Lactobacillus acidophilus* LA-5 (**c**,**d**) after applying BSG flours of SLE (**a**,**c**) and OHE (**b**,**d**) using three concentrations (2, 1.5 and 1% (*v/w*)). x Positive control—Glucose; ● Positive control—FOS; + 60% ethanol:water SLE or OHE_2% (*v/w*); - 60% ethanol:water SLE or OHE_1.5% (*v/w*); ● 60% ethanol:water SLE or OHE_1% (*v/w*); - 80% ethanol:water SLE or OHE_2% (*v/w*); 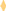 80% ethanol:water SLE or OHE_1.5% (*v/w*); ● 80% ethanol:water SLE or OHE_1% (*v/w*).

After GID simulation the BSG flours extracts were used to test prebiotic potential activity for four different strains of probiotic bacteria *Bifidobacterium animalis B0* (*B. animalis* B0), *Bifidobacterium animalis* spp. *lactis* BB12 (BB12), *Lacticaseibacillus casei* 01 (*L. casei* 01) and *Lactobacillus acidophilus* LA-5 (*L. acidophilus* LA-5) and three concentrations were used 2, 1.5 and 1% (*w/v*). In Figure 2 and Figure 3 it is possible to observe the growth curves of the probiotic bacteria.

With regard to *B. animalis* B0, it was possible to see that the samples induced growth on the microorganism. Although this happened, the growth induced by all concentrations tested did not present as high OD as the growth induced by the positive controls (glucose and FOS). Between extraction methods it was also possible to observe that there were almost no differences between the results obtained, although the 60% ethanol:water (*v/v*) sample at 2% (*v/w*) had a higher OD with OHE method. In both extraction methods the sample using 60% ethanol:water (*v/v*) at 1% (*v/w*) was the one that induced more growth in the microorganism.

In Figure 3 the results obtained for BB12 are also present. When looking at these results it was possible to observe that also here the samples induced growth in the microorganism, and here the OD values of the samples are similar to the ones obtained for the FOS positive control, although remaining smaller than the glucose positive control. It was also positive to observe that there were no differences between both extraction methods results. With this microorganism it was also the sample using 60% ethanol:water (*v/v*) at 1% (*v/w*) that induced more growth.

With regard to *L. casei* 01 (Figure 3) it was possible to observe that all the samples induced growth in the microorganism with some samples having OD values closed to the positive controls (glucose and FOS). Although this happened, it was also possible to observe that for both extractions methods tested (SLE and OHE) 60% and 80% ethanol:water (*v/v*) at 1% (*v/w*) was the concentration with lower OD values.

When observing the results of the samples on *L. acidophilus* LA-5 it was possible to observe that for all the tested concentrations in both extraction methods the OD values (growth induced) was similar and it was also similar to the FOS positive control; however, these values were smaller than the glucose positive control.

Although this was the first time BSG flours obtained from these two types of extraction (SLE and OHE) were used to evaluate the prebiotic activity potential of BSG, other studies [60,61] tested the prebiotic effect of BSG throughout the GID having positive results. Arena et al. (2014) [62] and Charalampopoulos, Pandiella, and Webb (2003) [63] all tested the prebiotic effect of compounds similar to BSG (barley, malt, wheat and other cereals) also with positive results. These results prove that the prebiotic bacteria used in these studies are able to utilise the compounds formed by the digestion of the BSG flours, thus inducing growth in the microorganisms tested, which can make of these residues an interesting application in the food industry.

### 3.4. Evolution of the Gut Microbiota Profile Groups

The BSG flours from both extractions used (SLE and OHE) were applied to human faeces and fermented during 48 h and, through the real-time PCR investigation on the four dominant phyla in the human gut microbiota, Firmicutes, *Lactobacillus* spp., *Enterococcus* spp., *Bacteroidetes* spp., *Bacteroides* spp., and *Bifidobacterium* spp., were studied the effect of the samples on human gut microbiota. Aliquots at 0, 12, 24 and 48 h were taken and analysed to understand the modulation of the microorganisms’ growth and metabolic activities during the gut fermentation. FOS was used as a positive control—a compound with prebiotic effect in gut microbiota [21]. In Table 4 are present the compositional average copy numbers obtained by RT-PCR of these main groups.

The dominant genera present were *Bacteroides* spp. and *Bifidobacterium* spp. and the subdominant being *Lactobacillus* spp. and *Enterococcus* spp. In healthy adults, the Gram-positive *Firmicutes* and Gram-negative *Bacteroidetes* are the main phyla in the gut microbiome and *Lactobacillus* spp. is usually present in lower numbers [15,21,64].

**Figure 4:**
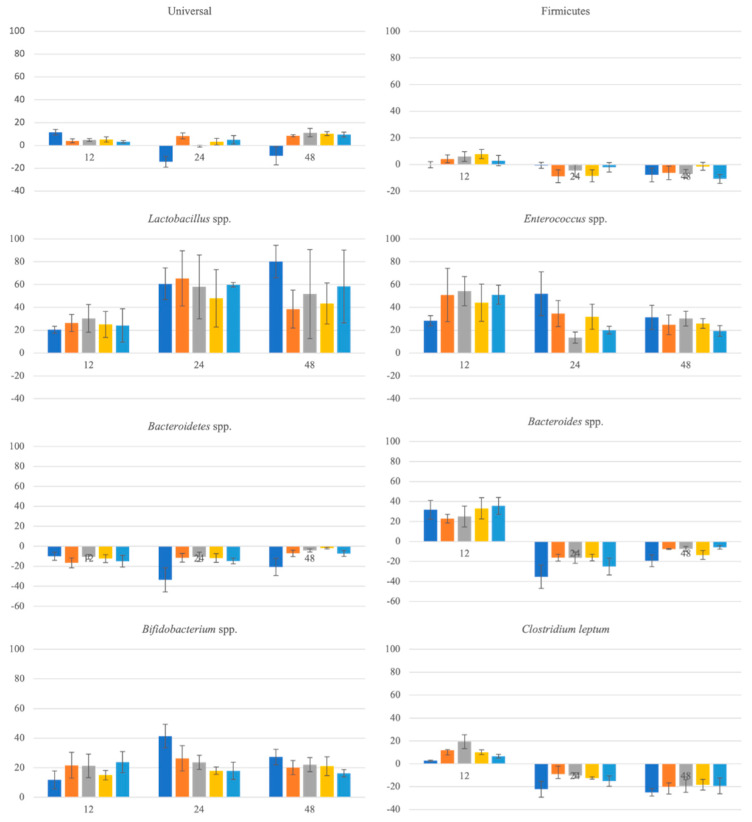
Evolution of the gut microbiota groups (relative differences to the negative control in %). ■—FOS; ■—60% ethanol:water SLE; ■—80% ethanol:water SLE; ■—60% ethanol:water OHE; ■—80% ethanol:water OHE.

In Figure 4 are present the relative differences (%) between microbiota groups of the tested samples and control faeces at 12, 24 and 48 h of fermentation. Overall, all samples of this study promoted a somewhat positive effect on the growth of the gut microbiota. There was an increment of the universal microorganisms over time when comparing with the control while FOS had a positive effect on the first 12 h being reversed at the 24 h and grew again in the last 48 h. It is worth noticing that FOS exerted a positive effect in all phyla with the exception of *Firmicutes* after 12 h, *Bacteroidetes* spp., and *Bacteroides* and *Clostridium leptum* after 12 h. These differences could be related to the no use of pH control in this in vitro faeccal fermentation study.

All BSG flours showed to have a positive effect on *Lactobacillus* spp. and *Bifidobacterium* spp., two bacterial groups associated with beneficial gut microbiota [15,65]. While with *Lactobacillus* spp. the growth was similar during the 48 h, with *Bifidobacterium* spp. at 24 h the growth was higher than at 12 h decreasing at the 48 h. All samples promoted growth of the probiotic strains, which mean they might act as prebiotics. However, all the samples had a negative effect for *Firmicutes* after the first 12 h. As this phyla represents several microorganisms as *Lactobacillus* spp. and *Clostridium leptum* it might be possible to conclude maybe these samples had a negative effect upon the other potential groups of this phyla that was higher than the positive effect they had upon *Lactobacillus* spp. These results are in accordance with the results obtained in the prebiotic study as there all the samples exerted a positive effect in all tested microorganisms.

With regard to *Bacteroidetes* spp., it was possible to conclude that as with FOS, all the samples had a negative effect. Both *Firmicutes* and *Bacteroidetes* are representative of a healthy microbiota [15].

It is worth noticing the result upon the *Enterococcus spp*. group where all the samples produced a positive effect upon this group even though the levels of these microorganisms decreased after 12 h, while FOS promoted growth at 24 h, decreasing again at 48 h. Two strains of *Enterococcus* spp. (*E. faecium* SF68^®^ and *E. faecalis* Symbio-flor^®^) have been known probiotics for a few decades. Probiotics of this genus showed to have effect on the gastrointestinal infectious burden and on the treatment of gastrointestinal infections and diarrhea [66,67]. However, constrained gut health is sometimes linked to this genus [68].

At 12 h, all the samples and FOS exerted a positive effect upon *Bacteroides* spp.; however, after this time all had a negative effect in this group. As with *Enterococcus* spp. this group is on one side associated with diarrhea, inflammatory bowel disease, and other intestinal dysfunctions, on the other side, it can be considered a probiotic as it has a potential role in the promotion of host health by regulation the intestinal redox levels or the production of important short chain fatty acids [69].

It was also possible to observe that while at 12 h all the samples and FOS had a positive effect upon the growth of *Clostridium leptum*; after this time all samples and FOS exerted a negative effect in this group.

**Figure 5:**
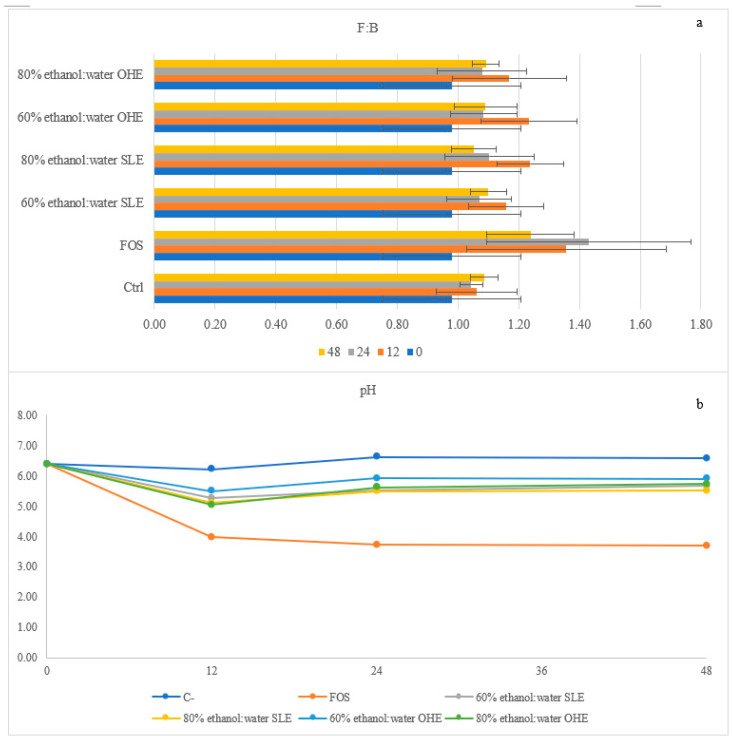
Firmicutes: Bacteroidetes (F:B) (**a**) ratio and variation of the pH (**b**) throughout fermentation of FOS and BSG flours with human microbiota.

In Figure 5a it is present the Firmicutes:Bacteroidetes (F:B) ratio, which involves two of the most abundant phyla in the human microbiota [15]. A healthy individual normally has a F:B ratio nearly 1:1 and significant alterations of this value might be associated with patological issues such, higher F:B ratios have been linked to obesity, while lower F:B ratios have been linked to type-II diabetes mellitus [70,71,72]. Higher the values, higher the content in *Firmicutes* in relation to the content of *Bacteroidetes*. In this study positive control (FOS) had higher values of this ratio throughout all the hours of the fermentation (when comparing to BSG samples). All BSG samples had a ratio higher than 1:1 throughout the 48 h of fermentation.

It is worth noting that even though two different type of extractions were used and two solvent ratios, the four types of BSG flours (SLE 60 and 80% ethanol:water (*v/v*) and OHE 60% and 80% ethanol:water (*v/v*)) demonstrated a similar behaviour throught the 48 h.

BSG samples were able to promote the growth of phyla of good microbiota and at the same time to control the growth of not so desirable genus as *Bacteroides* spp., and might be considered as an ingredient with functional properties and to be applied in functional foods.

Other studies reported the effect of BSG on gut microbiota. Niemi et al. (2013) [73] studied the effect of a lignin-rich fraction of BSG with gut microbiota. This fraction of BSG flour proved to not inhibit the growth of beneficial gut bacteria as lactobacilli and bifidobacteria, with some samples to enable the growth of bifidobacteria for longer time than the positive control. Sajib et al. (2018) [61] used BSG hydrolysates to assess their use by human microbiota. Results revealed that BSG hydrolysates were fermented similarly to the positive control. Reis et al., 2014 [19] studied if arabinoxylans from BSG had an effect on gut microbiota, proving that these arabinoxylans increase the growth of bifidobacteria populations. The results in this study are in accordance with the literature as the extraction residues of BSG were able to promote the growth of lactobacilli and bifidobacterial demonstrating that the use of these there is a relevant value added for these flours obtained from solid residues through extraction in the food industry an added value. However, this was the first study to use SLE and OHE and to assess the solid residues of these extractions on the gut microbiota.

### 3.5. Organic Acids and Sugar Profiles during Gut Microbiota Fermentation

The identification and quantification of short-chain fatty acids (SCFA) and other organic acids along the 48 h of fermentation was also made and the results are present in Table 4. Furthermore, in Figure 5b is represented the variations of pH along the fermentation. With regard to organic acids (Table 5), five were identified during the formation time (succinic, lactic, acetic, propionic and butyric acids) throughout all samples. Butyric acid was the one with higher concentration in all samples after 48 h, while lactic and butyric acids were the ones with higher concentrations in FOS after 48 h of fermentation (*p* < 0.05). Lactate was in fact the main metabolite produced during the entire fermentation of FOS, which can be related to the stimulatory effects of FOS on the *Bifidobacterium* spp. and *Lactobacillus* spp. validated with the gene analysis above. However, the accumulation of lactic acid when using FOS might mean that it was not used by as a substrate by bacteria and has this study was conducted without a pH control, the sharp decrease of pH in FOS may have not allowed the growth of lactate-using bacteria, leading to an accumulation of lactate during the fermentation [74,75]. Lactic acid was not detected before fermentation, meaning it was produced during fermentation as a result of carbohydrates metabolization. Moreover, regarding lactic acid, it was possible to observe in all samples an increased from 0 to 24 h followed by a decreased at 48 h. Acetate production was also stimulated under FOS presence and a similar pattern was followed by all four BSG samples (no statistical difference (*p* < 0.05) with the production of acetate increasing during the 48 h fermentation.

Butyrate is one of the most important SCFAs produced in the gut by eubacteria and clostridia as it has a positive effect in host health by having anti-inflammatory and regenerative effects, helping against colon cancer formation among others [15,70,75]. In this study, even though all the samples promoted the increase of this organic acid, the accumulation of butyrate was more pronounced for FOS while BSG samples had values proximate to the negative control. The same happened to Campos et al. (2020) [15] and Gullón et al. (2014) [76] having higher values of butyric acid for the positive control when comparing to pineapple flours and arabinooligosaccharides. Butyrate had its highest values at 48 h for FOS and 60 and 80% (ethanol:water) OHE, while for the other two samples (60 and 80% (ethanol:water) SLE) the highest values were at 24 h.

Acetate, propionate and butyrate are the most common SCFAs produced by intestinal bacteria through fermentation of non-digestible carbohydrates, although formate, isobutyrate and others can also be produced [15,77]. These three acids play an important role in the maintenance of intestinal homeostasis [78,79].

All samples promoted an increase in acetate levels having achieved a maximum concentration at 48 h. *Bifidobacterium* bacteria are one of the producers of acetate [79] and the acetate values can be explained by the growth increase of the *Bifidobacterium* group.

Interestingly enough, all samples promoted an increase in succinate levels having the maximum concentration at 24 h decreasing after that. This organic acid is normally associated with microbiota disturbances linked to poor gut health [80] and is also associated to the production of propionate, which is responsible for controlling appetite, preventing colon cancer, among other things [81]. Propionate levels follow a similar behaviour to succinate with all the samples increasing until 24 h decreasing after that.

The positive control (FOS) had a positive accumulation of SCFAs during the 48 h, while the BSG samples increase until 24 h but slightly decrease at the end of the 48 h. However, their total concentrations were higher than the negative control accumulation concentrations. These values are in accordance with the pH results. A high decrease of pH occurred for FOS and a decreased occurred for all the BSG samples, while for the positive control an increase of pH happened.

The higher value of SCFAs of all samples was obtained after 24 h (between 6.95 and 6.32 mg/mL). Between samples the differences between SCFAs concentration are almost inexistent and all maintained a similar behaviour.

To all of our knowledge, this was the first study using flours obtained from SLE and OHE extraction residues to evaluate the SFCAs production. However, other studies using BSG have published results about this interaction. Niemi et al. (2013) [73] studied the effect of a lignin-rich fraction of BSG on the production of SFCAs. In this study the main SFCAs increased with the fermentation and were higher than the negative control. Sajib et al. (2018) [61] used BSG hydrolysates to assess their use by human microbiota and here the pH of the samples decreased and the concentration of acetic, propionic and butyric acids increased and were higher than the negative control. Reis et al., 2014 [19] studied if arabinoxylans from BSG had an effect on gut microbiota and SFCAs production. These arabinoxylans had their pH reduced with fermentation and the production of SFCAs was higher in the samples than in the negative and positive control.

The sugar and organics acids content of the faeces samples were evaluated through HPLC and the results can be observed in Table 4. The human faeces of the five donors were supplemented with FOS as a positive control and concentrations of two sugars (saccharose and glucose) were found throughout the 48 h of fermentation. The negative control (with no supplementation) had residual amounts of sugars present.

When observing the results of the BSG flours there was no statistical difference (*p* < 0.05) between the two extractions tested (SLE and OHE) and the two solvent ratios used (60 and 80% ethanol:water (*v/v*) throughout the fermentation time. With regard to saccharose, at 12 h both 60% ethanol:water (*v/v*) SLE and OHE had higher results (statistically different (*p* < 0.05)). These two flours type also had the higher result of glucose at 48 h (statistically different (*p* < 0.05)). There was an increase in glucose concentration between the 12 h and 24 h, which was metabolised between the 24 h and 48 h of fermentation.

With regard to saccharose, there was an increase after 12 h of fermentation followed by a decreased after the 24 h of fermentation due to the consumption during the microbiota fermentation.

### 3.6. Polyphenols Profile during Gut Microbiota Fermentation

The polyphenols were evaluated through HPLC for 0, 12, 24 and 48 h of fermentation and the results are present in Table 6. The results showed that vanillic, ferulic (hydroxycinnamic acids) and 4-hydroxybenzoic (hydroxybenzoic acid) acids and catechin (flavonoid) were identified in all samples at 12, 24 and 48 h. Syringic acid and 4-hydroxiferulic acid were also identified but not quantified.

After 12 h of fermentation the 60% ethanol:water (*v/v*) SLE presented higher concentration of ferulic acid (3.01 mg/100 g BSG), while the polyphenol with the highest concentrations throughout the fermentation was catechin, being present in higher concentrations in 60% ethanol:water (*v/v*) OHE (between 13.52 and 23.72 mg/100 g BSG). When using 60% ethanol:water SLE (*v/v*) the phenolic compounds identified had their concentration reduced throughout the 48 h with the exception of ferulic acid that increased at 48 h and 4-hydroxybenzoic acid that increased at 24 h. Ferulic acid had its concentration increased always at 48 h in all the extractions used. 4-hydroxybenzoic acid increased its concentration always at 24 h, then reducing at 48 h.

Overall, after 48 h of fermentation the concentration of the polyphenols decreased in all the samples studied except for ferulic acid that increased at that time. These results show that the antioxidant potential of BSG flours can be maintained throughout the GID simulation as some polyphenols are still complexed with dietary fibre, being after released and utilized by human microbiota. Insoluble-bound phenolics will not be absorbed in the small intestine as they are bound to macromolecules as cellulose, hemicellulose, protein and pectin, which are insoluble macromolecules. Ferulic and vanillic acid are hydroxycinnamic acids and 4-hydroxybenzic acid a hydroxybenzoic acid that normally are linked to insoluble dietary fibre, which means that the human gut microbiota was able to release these compounds from insoluble fibres and other molecules. These three phenolic acids have been reported to be present in the insoluble-bound form in cereals as barley, wheat oat, rice, between others. Catechin, a flavonoid, can be present in cereals in the soluble and insoluble-bound form and it has been reported the presence of catechin in the insoluble-bound form in cereals corn, wheat and barley [15,82].

Campos et al. (2020) [15] and Quirós-Sauceda et al. (2014) [83] described the interaction of the human intestinal microbiota with dietary fibre contributing to the released of polyphenols in the intestinal lumen and being absorbed by gut epithelial cells. This leads to the polyphenols that were not absorbed to remaim in the colonic tissue and scavenging free radicals radical and preventing the dietary fibre pro-oxidants effects. The human intestinal microbiota (*Bifidobacterium* spp. and *Lactobacillus* spp) will releasee enzymes that will disrupt the cell wall matrix or the covalent bonds of bound phenolics, releasing these phenolics [83].

The antioxidant dietary fibre is capable of having positive effects on lipid metabolism, total cholesterol and others, and to increase the antioxidant activity in the large intestine. This might have a positive effect in the prevention of some form of cancer (by preventing the growth of cancer inducing microorganisms) and chronic diseases [82,84,85].

Again, this was the first study using BSG flours obtained after SLE and OHE extraction with gut microbiota. However, Niemi et al. (2013) [73] studied the effect of a lignin-rich fraction of BSG with gut microbiota. In this study, the different polyphenols were identified, such as 4-methylcatechol, 3,4-dihydroxyphenylacetic, p-coumaric and ferulic acids and contrary to what happened in this study, the concentration of ferulic acid declined during the fermentation. This might have happened as the lignin was only partially degraded by the colon and the metabolites present were released slower and differently as in what happened in this study.

## 4. Conclusions

For the first time was studied the impact of GID on BSG flours obtained from solid residues of SLE and OHE and their influence on human fecal microbiota modulation. Overall, the extracts, independent of the extraction method increase the total phenolic content and the antioxidant activity throughout GID simulation. No relative difference between the two methods of extraction was observed so, industrially, the decision to opt between SLE or OHE would only be left to the costs, eco-friendly and advantages of one or the other.

In the intestinal phase, a cleavage of soluble dietary fibre happened leading to the release of the phenolic compounds with higher size, being the antioxidant activity higher in this stage for all the samples.

The polyphenol profile was similar for all the extracts tested being vanillic, ferulic, *p*-coumaric, 4-hydroxybenzoic acids, vanillin and catechin identified and quantified. However, the concentration of these compounds decreased or was not detected throughout the GID, which might mean that the GID degrades the molecules making them lose their activity.

The BSG flours also proved to induce the growth of probiotic bacteria making of these residues good candidates to be a prebiotic.

BSG flours were tested by human gut bacteria maintaining or enhancing the growth of specific microorganisms. All the samples positively promoted the growth of *Lactobacillus* spp. and *Bifidobacterium* spp. most relevantly. The SCFAs content was also evaluated throughout human gut microbiota fermentation with all the samples inducing the production of these compounds with higher concentrations than the negative control. The phenolic content of each polyphenol in the human gut microbiota fermentation was also evaluated being identified vanillic, ferulic, 4-hydroxybenzoic acids and catechin throughout the 48 h of the fermentation. The presence of these compounds after the human gut microbiota fermentation might show a connection between these molecules and insoluble dietary fibre.

In conclusion, all the samples analyzed in this study can be a controlled released system of polyphenols with antioxidant activity combining prebiotic potential. These BSG flours with all these biological properties exert a positive effect in each stage of the GID, as well as in the human gut.

## Figures and Tables

**Table 1:** Primer sequences and real-time PCR conditions used for gut microbiota analysis.

Target Group	Maximum Growth Rate (µmax.h^−1^)			
	Primer Sequence (5′-3′)	Genomic DNA Standard	PCR Product Size (bp)	AT (°C)
*Universal*	AAA CTC AAA GGA ATT GAC GG ACR RCA CGA GCT GAC	*Bacteroides vulgatus * ATCC 8482 (DSMZ 1447)	180	45
*Firmicutes*	ATG TGG TTT AAT TCG AAG CA AGC TGA CGA CAA CCA TGC AC	*Lactobacillus gasseri* ATCC 33,323 (DSMZ 20243)	126	45
*Enterococcus* spp.	CCC TTA TTG TTA GTT GCC ATC ATT ACT CGT TGT ACT TCC CT TGT	*Enterococcus gilvus* ATCC BAA-350 (DSMZ 15689)	144	45
*Lacctobacillus* spp.	GAG GCA GCA GTA GGG AAT CTT C GGC CAG TTA CTA CCT CTA TCC TTC TTC	*Lactobacillus gasseri* ATCC 33,323 (DSMZ 20243)	126	55
*Bacteroidetes*	CAT GTG GTT TAA TTC GAT GAT AGC TGA CGA CAA CCA TGC AG	*Bacteroides vulgatus* ATCC 8482 (DSMZ 1447)	126	45
*Bacteroides* spp.	ATA GCC TTT CGA AAG RAA GAT CCA GTA TCA ACT GCA ATT TTA	*Bacteroides vulgatus* ATCC 8482 (DSMZ 1447)	495	45
*Bifidobacterium* spp.	CGC GTC YGG TGT GAA AG CCC CAC ATC CAG CAT CCA	*Bifidobacterium longum* subsp. *Infantis* ATCC 15,697 (DSMZ 20088)	244	50
*Clostridium leptum*	GCA CAA GCA GTG GAG T CTT CCT CCG TTT TGT CAA	*Clostridium leptum* ATCC 29,065 (DSMZ 753)	239	45

AT—annealing temperature; bp—base pairs; PCR—polymerase chain reaction.

**Table 2:** Concentration of total phenolic compounds and individual phenolic compounds present in BSG flours obtained after extraction using 60% ethanol:water and 80% ethanol:water (SLE) and 60% ethanol:water and 80% ethanol:water (OHE) of, for the non-digested (initial) and for the three stages of the GID (oral, stomach and intestine) expressed as mg/100 g BSG as well as the total phenolic compounds (mg gallic/g BSG) and bioaccessibility index (mean ± s.d.).

Type of Extraction mg/100g BSG	Stage	Total Phenolic Compounds (mg Gallic Acid/g BSG)	Vanillic Acid	% B	Ferulic Acid	% B	*p*-Coumaric Acid	% B	4-Hydroxybenzoic Acid	% B	Catechin	% B	Vanillin	% B
60% ethanol:water (SLE)	Initial	15.66 ± 2.08 ^A,a^	2.22 ± 0.00 ^A,a^	-	1.89 ± 0.06 ^D,a^	-	1.80 ± 0.05 ^B,a^	-	3.47 ± 0.08 ^A,a^	-	10.38 ± 0.14 ^A,a^	-	2.30 ± 0.01 ^B,a^	
	Mouth	2.77 ± 0.07 ^A,d^	N.D.	-	0.66 ± 0.00 ^C,b^	35.00	N.D.	-	0.92 ± 0.00 ^b^	26.60	4.38 ± 0.10 ^B,b^	41.18	N.D.	
	Stomach	3.84 ± 0.16 ^C,c^	N.D.	-	N.D.	-	N.D.	-	0.06 ± 0.00 ^c^	1.86	3.68 ± 0.33 ^A,c^	35.50	N.D.	
	Intestine	7,20 ± 0.29 ^B,b^	N.D.	-	N.D.	-	N.D.	-	N.D.	-	0.81 ± 0.12 ^A,d^	7.80	N.D.	-
80% ethanol:water (SLE)	Initial	13.77 ± 0.39 ^A,a^	2.06 ± 0.20 ^AB,a^	-	1.75 ± 0.06 ^C,a^	-	3.08 ± 0.05 ^A,a^	-	3.39 ± 0.06 ^AB,a^	-	8.03 ± 0.70 ^B,a^	-	2.45 ± 0.03 ^A,a^	
	Mouth	2.31 ± 0.00 ^B,d^	N.D.	-	0.97 ± 0.01 ^B,b^	55.45	0.28 ± 0.01 ^A,b^	9.09	N.D.	-	6.00 ± 0.34 ^A,b^	74.78	N.D.	
	Stomach	5.74 ± 0.03 ^A,c^	N.D.	-	N.D.	-	N.D.	-	N.D.	-	2.08 ± 0.00 ^B,c^	25.93	N.D.	
	Intestine	6.27 ± 0.45 ^D,b^	N.D.	-	N.D.	-	N.D.	-	N.D.	-	0.26 ± 0.01 ^C,d^	3.28	N.D.	-
60% ethanol:water (OHE)	Initial	10.91 ± 0.19 ^B,a^	1.85 ± 0.27 ^BC,a^	-	1.98 ± 0.02 ^B,a^	-	0.17 ± 0.02 ^D,b^	-	3.33 ± 0.00 ^B,a^	-	7.21 ± 0.51 ^B,a^	-	2.29 ± 0.00 ^B,a^	
	Mouth	2.23 ± 0.07 ^AB,d^	N.D.	-	0.97 ± 0.24 ^AB,b^	48.86	0.28 ± 0.00 ^A,a^	170.64	N.D.	-	1.96 ± 0.02 ^C,b^	27.21	0.97 ± 0.00 ^A,d^	42.57
	Stomach	5.66 ± 0.09 ^A,c^	N.D.	-	N.D.	-	N.D.	-	N.D.	-	0.94 ± 0.01 ^C,c^	13.03	1.06 ± 0.00 ^B,c^	46.25
	Intestine	7.39 ± 0.03 ^C,b^	N.D.	-	N.D.	-	N.D.	-	N.D.	-	0.29 ± 0.01 ^B,d^	4.06	1.37 ± 0.00 ^B,b^	59.92
80% ethanol:water (OHE)	Initial	11.51 ± 0.59 ^B,a^	1.62 ± 0.02 ^C,a^	-	2.30 ± 0.30 ^A,a^	-	0.92 ± 0.08 ^C,a^	-	3.34 ± 0.01 ^B,a^	-	6.62 ± 0.04 ^C,a^	-	2.27 ± 0.01 ^C,a^	
	Mouth	2.23 ± 0.03 ^B,d^	N.D.	-	1.08 ± 0.11 ^AB,b^	47.21	0.25 ± 0.01 ^B,b^	27.03	N.D.	-	4.26 ± 0.02 ^B,b^	64.37	0.97 ± 0.00 ^A,d^	42.81
	Stomach	4.16 ± 0.67 ^B,c^	N.D.	-	0.96 ± 0.15 ^A,b^	88.62	0.22 ± 0.00 ^A,b^	24.11	N.D.	-	2.06 ± 0.02 ^B,c^	31.13	1.61 ± 0.00 ^A,b^	71.15
	Intestine	8.36 ± 0.05 ^A,b^	N.D.	-	N.D.	-	0.05 ± 0.01 ^A,c^	5.41	N.D.	-	0.26 ± 0.01 ^C,d^	4.00	1.52 ± 0.00 ^A,c^	66.90

Different letters mean significant differences, determined by ANOVA and Tukey’s post-hoc test (*p* < 0.05). The capital letters indicate the differences among the 60% ethanol:water and 80% ethanol:water (*v/v*) (SLE and OHE) for polyphenols concentration at the same GID phase, and the lowercase letters indicate the differences between GID phases for each individual polyphenol in each extract. N.D. = Non detected.

**Table 3:** Percentages of differences between the different phases of the gastrointestinal tract (GID) simulation of brewer’s spent grain (BSG) flours.

Phases of the GID	Type of Extraction	ABTS (%)	ORAC (%)	Folin-Ciocalteu (%)
Initial/Oral	60% ethanol:water SLE	47.04	81.15	81.04
	80% ethanol:water SLE	67.95	79.03	83.80
	60% ethanol:water OHE	58.70	88.65	79.60
	80% ethanol:water OHE	64.97	81.90	80.63
Oral/Gastric	60% ethanol:water SLE	−16.70	−122.13	−29.34
	80% ethanol:water SLE	−11.50	−63.12	−157.46
	60% ethanol:water OHE	−46.58	−348.47	−154.47
	80% ethanol:water OHE	−63.46	−193.37	−86.58
Gastric/Duodenal	60% ethanol:water SLE	−47.30	−102.98	−87.46
	80% ethanol:water SLE	−87.38	−153.09	−9.12
	60% ethanol:water OHE	−35.95	−81.97	−30.53
	80% ethanol:water OHE	−40.97	−70.94	−101.01

**Table 4:** Fecal microbiota composition of volunteer participants.

Division (Genus)	Number of Copies (n = 5) a
Universal	7.55 ± 0.37
Firmicutes	4.76 ± 0.19
*Enterococcus* spp.	1.76 ± 0.82
*Lactobacillus* spp.	2.93± 0.93
*Bacteroidetes* spp.	5.05 ± 0.98
*Bacteroides* spp.	3.73 ± 0.42
*Bifidobacterium* spp.	4.24 ± 0.57
*Clostridium leptum*	4.97 ± 0.25
F:B ratio	0.98 ± 0.21

a Values are presented as mean ± SD and expressed as log10 16S rRNA gene copies per 20 ng of DNA.

**Table 5:** Concentration of organic acids (succinic, lactic, acetic, propionic and butyric) and sugars (saccharose and glucose) throughout fermentation of FOS and BSG flours with human microbiota (*w/v*).

Organic Acids/Sugars (*w/v*)	Time (h)	Ctrl	FOS	60% Ethanol:Water (SLE)	80% Ethanol:Water (SLE)	60% Ethanol:Water (OHE)	80% Ethanol:Water (OHE)
Succinic acid	0	0.45 ± 0.26 ^A,b^	0.45 ± 0.26 ^A,b^	0.45 ± 0.26 ^A,c^	0.45 ± 0.26 ^A,c^	0.45 ± 0.26 ^A,c^	0.45 ± 0.26 ^A,c^
	12	1.93 ± 1.18 ^A,a^	1.85 ± 0.92 ^A,a^	1.32 ± 0.07 ^A,b^	1.29 ± 0.10 ^A,b^	1.40 ± 0.07 ^A,a^	1.48 ± 0.29 ^A,b^
	24	1.18 ± 0.56 ^B,a^	1.87 ± 0.62 ^B,a^	1.97 ± 0.22 ^B,a^	2.04 ± 0.15 ^B,a^	1.77 ± 0.71 ^AB.a^	2.07 ± 0.09 ^B,a^
	48	0.74 ± 0.71 ^B,b^	2.03 ± 0.85 ^A,a^	1.27 ± 0.08 ^B,b^	1.19 ± 0.09 ^B,b^	1.18 ± 0.06 ^B,b^	1.20 ± 0.04 ^B,b^
Lactic acid	0	ND	ND	ND	ND	ND	ND
	12	1.21 ± 0.93 ^B,a^	4.04 ± 2.74 ^A,a^	0.23 ± 0.03 ^C,a^	0.25 ± 0.03 ^C,a^	0.28 ± 0.06 ^C,a^	0.30 ± 0.15 ^C,a^
	24	0.34 ± 0.14 ^B,b^	5.19 ± 1.40 ^A,a^	0.34 ± 0.04 ^B,a^	0.33 ± 0.06 ^B,a^	0.33 ± 0.09 ^B,a^	0.36 ± 0.14 ^B,a^
	48	ND	5.49 ± 2.14 ^A,a^	0.24 ± 0.06 ^B,a^	0.28 ± 0.11 ^B,a^	0.27 ± 0.04 ^B,a^	0.27 ± 0.06 ^B,a^
Acetic acid	0	0.16 ± 0.04 ^A,b^	0.16 ± 0.04 ^A,b^	0.16 ± 0.04 ^A,c^	0.16 ± 0.04 ^A,c^	0.16 ± 0.04 ^A,b^	0.16 ± 0.04 ^A,c^
	12	0.65 ± 0.27 ^B,a^	1.64 ± 0.65 ^A,a^	0.97 ± 0.15 ^B,b^	0.80 ± 0.12 ^B,b^	0.94 ± 0.12 ^B,ab^	0.96 ± 0.05 ^B,b^
	24	0.74 ± 0.23 ^C,a^	1.48 ± 0.59 ^A,a^	1.12 ± 0.10 ^AB,ab^	1.13 ± 0.14 ^AB,a^	1.10 ± 0.12 ^BC,ab^	1.12 ± 0.11 ^AB,ab^
	48	0.69 ± 0.25 ^B,a^	2.29 ± 1.26 ^A,a^	1.22 ± 0.08 ^B,a^	1.24 ± 0.08 ^B,a^	1.38 ± 0.20 ^B,a^	1.30 ± 0.14 ^B,a^
Propionic acid	0	0.34 ± 0.09 ^A,c^	0.34 ± 0.09 ^A,b^	0.34 ± 0.09 ^A,c^	0.34 ± 0.09 ^A,b^	0.34 ± 0.09 ^A,c^	0.34 ± 0.09 ^A,c^
	12	1.59 ± 0.32 ^A,a^	1.47 ± 0.60 ^A,a^	1.06 ± 0.36 ^A,b^	0.97 ± 0.61 ^A,a^	1.01 ± 0.13 ^A,b^	1.00 ± 0.41 ^A,b^
	24	0.81 ± 0.27 ^A,b^	1.95 ± 0.88 ^A,a^	1.31 ± 0.17 ^A,a^	1.24 ± 0.09 ^A,a^	1.26 ± 0.09 ^A,a^	1.38 ± 0.16 ^A,a^
	48	0.49 ± 0.18 ^C,c^	1.86 ± 0.79 ^A,a^	1.29 ± 0.14 ^AB,a^	1.23 ± 0.10 ^ABC,a^	1.25 ± 0.10 ^ABC,a^	1.22 ± 0.06 ^ABC,a^
Butyric acid	0	1.41 ± 0.25 ^A,b^	1.41 ± 0.25^A,b^	1.41 ± 0.25 ^A,b^	1.41 ± 0.25 ^A,a^	1.41 ± 0.25 ^A,b^	1.41 ± 0.25 ^A,b^
	12	1.55 ± 0.63 ^A,ab^	2.29 ± 0.99 ^A,a^	1.63 ± 0.27 ^A,b^	1.48 ± 0.27 ^A,a^	1.51 ± 0.40 ^A,ab^	1.54 ± 0.27 ^A,b^
	24	2.24 ± 0.67 ^A,a^	2.23 ± 0.66 ^AB,a^	2.21 ± 0.36 ^AB,a^	1.99 ± 0.34 ^B,a^	1.85 ± 0.16 ^B,a^	1.87 ± 0.42 ^B,a^
	48	1.83 ± 0.94 ^A,a^	2.70 ± 1.43 ^AB,a^	2.07 ± 0.18 ^B,a^	1.97 ± 0.62 ^AB,a^	2.08 ± 0.60 ^AB,a^	2.01 ± 0.25 ^AB,a^
Total organic acids	0	2.37 ± 0.64	2.37 ± 0.64	2.37 ± 0.64	2.37 ± 0.64	2.37 ± 0.64	2.37 ± 0.64
	12	5.92 ± 3.34	11.29 ± 5.89	5.21 ± 0.78	4.79 ± 1.03	5.14 ± 0.72	5.18 ± 1.22
	24	5.31 ± 1.87	12.72 ± 4.35	6.95 ± 0.89	6.73 ± 0.79	6.32 ± 1.16	6.81 ± 0.91
	48	3.75 ± 2.08	14.32 ± 6.46	5.99 ± 0.61	5.82 ± 1.05	5.91 ± 0.98	5.75 ± 0.58
Saccharose	0	1.02 ± 0.07 ^A,a^	1.02 ± 0.07 ^A,c^	1.02 ± 0.07 ^A,b^	1.02 ± 0.07 ^A,b^	1.02 ± 0.07 ^A,b^	1.02 ± 0.07 ^A,b^
	12	0.19 ± 0.14 ^D,b^	1.04 ± 0.03 ^C,c^	1.67 ± 0.10 ^B,a^	1.55 ± 0.07 ^B,a^	1.51 ± 0.09 ^B,a^	1.53 ± 0.11 ^Ba,^
	24	0.07 ± 0.01 ^C,c^	1.51 ± 0.09 ^B,a^	1.61 ± 0.12 ^A,a^	1.53 ± 0.05 ^B,a^	1.61 ± 0.07 ^A,a^	1.58 ± 0.11 ^AB,a^
	48	0.05 ± 0.01 ^C,c^	1.28 ± 0.05 ^A,b^	1.09 ± 0.04 ^B,b^	1.06 ± 0.06 ^B,b^	1.07 ± 0.03 ^B,b^	1.13 ± 0.25 ^AB,b^
Glucose	0	0.51 ± 0.09 ^A,a^	0.51 ± 0.09 ^A,c^	0.51 ± 0.09 ^A,a^	0.51 ± 0.09 ^A,a^	0.51 ± 0.09 ^A,a^	0.51 ± 0.09 ^A,a^
	12	0.06 ± 0.00 ^C,b^	2.06 ± 0.88 ^A,b^	0.37 ± 0.04 ^B,b^	0.38 ± 0.04 ^B,b^	0.34 ± 0.02 ^B,b^	0.38 ± 0.03 ^B,c^
	24	0.06 ± 0.01 ^C,b^	2.51 ± 0.08 ^A,a^	0.49 ± 0.03 ^B,a^	0.45 ± 0.05 ^B,a^	0.47 ± 0.04 ^B,a^	0.43 ± 0.05 ^B,b^
	48	0.03 ± 0.01 ^D,c^	2.01 ± 0.09 ^A,b^	0.34 ± 0.06 ^B,b^	0.19 ± 0.05 ^C,c^	0.31 ± 0.07 ^BC,b^	0.17 ± 0.05 ^C,d^

Different letters indicate significant differences (*p* < 0.05). The capital letters indicate the differences among the Ctrl, FOS, 60% ethanol:water and 80% ethanol:water (*v/v*) (SLE and OHE) for organic acids and sugars concentration at the same time (same row), and the lowercase letters indicate the differences for the same sample over time for each organic acid and sugars concentration (same column within an organic acid). Data represent the mean ± SD of five independent assays.

**Table 6:** Concentration of polyphenols throughout fermentation of BSG flours with human microbiota (mg/100 g).

Type of Extraction (mg/100 g BSG)	Stage	Vanillic Acid	Ferulic Acid	4-Hydroxybenzoic Acid	Catechin	*p*-Coumaric	Vanillin
60% ethanol:water (SLE)	0	N.D.	N.D.	N.D.	N.D.	N.D.	N.D.
	12	1.36 ± 0.75 ^A,a^	3.01 ± 0.51 ^A,a^	1.98 ± 0.02 ^A,b^	21.07 ± 1.61 ^AB,a^	N.D.	N.D.
	24	1.03 ± 0.58 ^A,a^	1.03 ± 0.44 ^A,c^	6.14 ± 1.16 ^A,a^	17.57 ± 2.51 ^ab^	N.D.	N.D.
	48	0.82 ± 0.46 ^A,a^	2.28 ± 0.35 ^A,b^	0.82 ± 0.34 ^A,c^	12.31 ± 3.64 ^b^	N.D.	N.D.
80% ethanol:water (SLE)	0	N.D.	N.D.	N.D.	N.D.	N.D.	N.D.
	12	1.50 ± 0.81 ^A,a^	2.43 ± 0.19 ^AB,a^	1.88 ± 0.26 ^A,b^	16.61 ± 2.80 ^B,a^	N.D.	N.D.
	24	1.20 ± 0.68 ^A,a^	0.93 ± 0.32 ^A,b^	6.01 ± 1.37 ^A,a^	19.93 ± 4.01 ^a^	N.D.	N.D.
	48	0.75 ± 0.41 ^A,a^	2.44 ± 0.28 ^A,a^	1.39 ± 1.17 ^A,b^	14.89 ± 4.97 ^a^	N.D.	N.D.
60% ethanol:water (OHE)	0	N.D.	N.D.	N.D.	N.D.	N.D.	N.D.
	12	1.43 ± 0.78 ^A,a^	2.29 ± 0.22 ^AB,a^	1.80 ± 0.26 ^A,b^	23.72 ± 3.27 ^AB,a^	N.D.	1.72 ± 1.40 ^A,a^
	24	1.06 ± 0.61 ^A,a^	1.00 ± 0.45 ^A,b^	8.00 ± 1.27 ^A,a^	18.66 ± 2.27 ^ab^	N.D.	0.14 ± 0.08 ^A,b^
	48	0.91 ± 0.57 ^A,a^	2.17 ± 0.45 ^AB,a^	0.89 ± 0.37 ^A,b^	13.52 ± 4.90 ^b^	N.D.	N.D.
80% ethanol:water (OHE)	0	N.D.	N.D.	N.D.	N.D.	N.D.	N.D.
	12	1.44 ± 0.77 ^A,a^	2.14 ± 0.21 ^B,a^	1.88 ± 0.54 ^A,b^	16.26 ± 5.39 ^B,a^	0.27 ± 0.13 ^A,a^	2.20 ± 0.78 ^A,a^
	24	1.03 ± 0.57 ^A,a^	1.07 ± 0.35 ^A,c^	7.68 ± 0.92 ^A,a^	22.01 ± 4.91 ^a^	N.D.	0.18 ± 0.07 ^A,b^
	48	0.91 ± 0.50 ^A,a^	1.60 ± 0.14 ^B,b^	0.67 ± 0.14 ^A,c^	11.23 ± 1.53 ^b^	N.D.	N.D.

Different letters mean significant differences (*p* < 0.05). The capital letters indicate the differences among the 60% ethanol:water and 80% ethanol:water (*v/v*) (SLE and OHE) for polyphenols concentration at the same time, and the lowercase letters indicate the differences between GID phases for each individual polyphenol in each extract. N.D = Non detected.

## Data Availability

Data is contained within the article.
